# Machine learning-based prediction of 1-year mortality using nutritional and inflammatory factors for type A acute aortic dissection with malperfusion

**DOI:** 10.3389/fcvm.2025.1539267

**Published:** 2025-09-29

**Authors:** Yanda Zhang, David Marimekala, Hang Xing, Jing Yuan, Bo Zhang, Yi Song, Ting Wang, Bo Zhang, Long Wang

**Affiliations:** ^1^ Division of Cardiovascular Surgery, Henan Provincial Chest Hospital, Affiliated Chest Hospital of Zhengzhou University, Zhengzhou, Henan, China; ^2^ Division of Cardiothoracic Surgery, Rhode Island Hospital, Alpert Medical School of Brown University, Providence, RI, United States; ^3^ Department of Ultrasound, The First Affiliated Hospital of Zhengzhou University, Zhengzhou, Henan, China; ^4^ Department of Radiation Oncology, The Affiliated Cancer Hospital of Zhengzhou University, Zhengzhou, Henan, China; ^5^ Department of Pathology, Affiliated Hospital of Nantong University, Nantong University, Nantong, Jiangsu, China

**Keywords:** acute aortic dissection, artificial intelligence, inflammatory factor, nutritional factor, mortality

## Abstract

**Background:**

Acute aortic dissection is a life-threatening condition, and malperfusion significantly exacerbates the prognosis of patients diagnosed with type A Acute aortic dissection (ATAAD). Current risk assessment tools often fail to consider the impact of nutritional and inflammatory factors, limiting their predictive accuracy. The aim of this study was to develop a machine learning model that integrates nutritional and inflammatory indices to predict 1-year mortality in ATAAD patients with malperfusion.

**Methods:**

This retrospective study included 433 ATAAD patients with malperfusion from Henan Provincial Chest Hospital (August 2020 to June 2023). Four machine learning models—logistic regression, XGBoost, random forest, and deep neural network—were developed to predict 1-year mortality using inflammatory and nutritional laboratory values, indices, and other clinical variables. Model training employed stratified 5-fold cross-validation and SMOTE for imbalanced data. The area under the receiver operating characteristic (ROC AUC) and other performance metrics were used to evaluate model efficacy, while SHAP values were computed to interpret feature importance.

**Results:**

Among 433 ATAAD patients with malperfusion, the random forest model used inflammatory and nutritional laboratory values to achieve the highest discrimination (AUC = 0.8242, 95% CI 0.7095–0.9219), while the XGBoost model performed best with inflammatory and nutritional indices (AUC = 0.7334, 95% CI 0.6115–0.8488). Calibration curves and Brier scores indicated good agreement between predicted and observed outcomes. Decision curve analysis demonstrated consistent net benefit for random forest and XGBoost models across clinically relevant threshold probabilities. Feature importance and SHAP analyses identified albumin, platelet count, total cholesterol, and C-reactive protein as consistently influential predictors.

**Conclusion:**

Nutritional and inflammatory factors significantly contribute to the 1-year mortality risk of ATAAD patients with malperfusion. Machine learning models that incorporate these factors, particularly random forest and XGBoost, can effectively stratify patient risk and support clinical decision-making. These findings underscore the importance of a comprehensive approach to risk assessment that includes metabolic and inflammatory markers to enhance patient outcomes and guide personalized interventions.

## Introduction

Acute aortic dissection (AAD) is a critical and potentially fatal condition that demands immediate diagnosis and treatment. The estimated occurrence rate of AAD is approximately 5–30 cases per 1 million individuals annually (1). AAD results from a tear in the layers of the aortic wall, and if left untreated, mortality rates can increase to 50% within the initial 48 h (2). Most AADs involve the aortic arch and occur due to a tear where the aorta exits from the heart, and type A AADs (ATAAD) account for 70%–75% of all aortic dissections (3). Malperfusion occurs in approximately one-third of patients with AAD (4–7); furthermore, malperfusion has a substantial negative impact on patient prognosis by compromising blood flow to critical organs and tissues, significantly influencing both short- and long-term outcomes for affected individuals (8).

Studies have demonstrated that patients experiencing malperfusion have a significantly higher in-hospital mortality rate (21.5% vs. 5.7%) than to those without malperfusion (9). However, the specific 1-year mortality rate for ATAAD patients with malperfusion is not explicitly known. Various risk factors such as age, genetic predisposition, hypertension, and other comorbidities can increase a patient's likelihood of developing ATAAD and exacerbate their prognosis (10). Prompt recognition of ATAAD and appropriate management of malperfusion are crucial to improving outcomes for these critically ill patients.

Traditional risk assessment tools, such as the Stanford classification and the DeBakey classification, have limitations in predicting malperfusion in ATAAD patients. These traditional risk stratification models often overlook the role of nutritional and inflammatory factors, which are crucial in the pathophysiology of ATAAD. Inflammatory factors are involved in the body's response to trauma and indicate the state of lymphocytes, neutrophils, and other immune responses (11). These immune responses increase the expression of proteases and reactive oxygen species (ROS), which lead to increased apoptosis of smooth muscle cells in the aortic artery and further medial degradation (12). Recent studies have shown that nutritional and inflammatory factors are vital to the development and progression of ATAAD (13, 14). However, there is a lack of machine learning models that use these factors to accurately predict the 1-year mortality rate for ATAAD patients with malperfusion.

Identifying predictive indicators of 1-year mortality is essential for enhancing patient outcomes, since these patients require vigilant monitoring even after successful interventions. This study aimed to develop a machine learning model that incorporates nutritional and inflammatory factors to predict 1-year mortality in ATAAD patients with malperfusion. By integrating these critical factors, this study seeks to refine risk assessment and support targeted post-treatment care in ATAAD patients.

## Methods

### Data set

#### Patients

**Figure 1:**
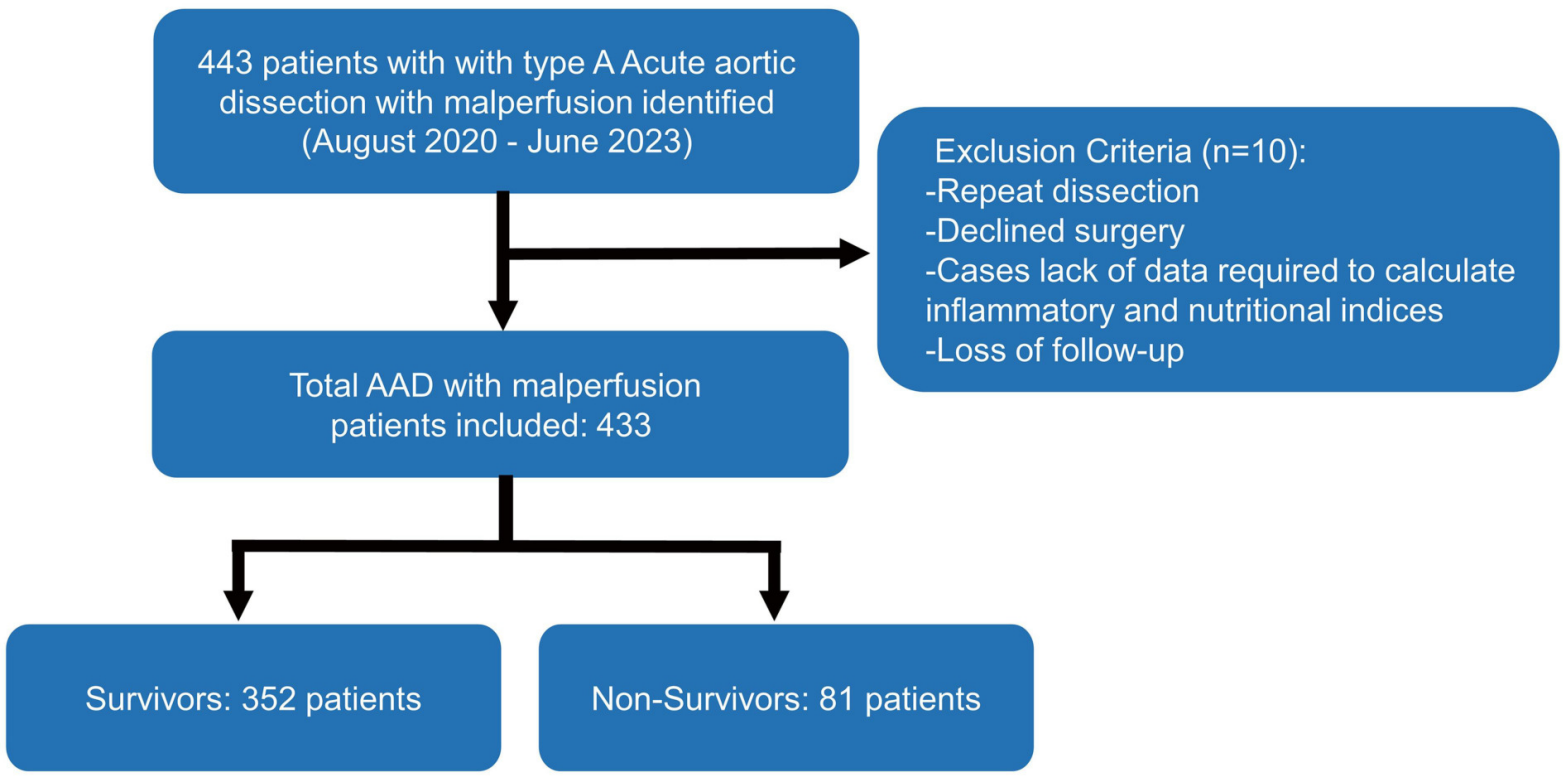
Flow chart of patient enrollment.

The study included 433 ATAAD patients with malperfusion from Henan Provincial Chest Hospital from August 2020 to June 2023. Approval for the study was obtained from the Henan Provincial Chest Hospital Ethics Committee [Reg. No. 2024 (09-16)]; since the study was retrospective, individual informed consent was waived. On the basis of the Diagnosis of Aortic Dissection published by the European Society of Cardiology (ESC) and the Stanford criterion, ATAAD was diagnosed via symptoms, physical examination, transthoracic echocardiography, and computed tomography angiography (CTA). Malperfusion was diagnosed in patients on the basis of clinical symptoms (altered consciousness, paralysis, melena, abdominal pain, tenderness to palpation, loss of sensory or motor function of the lower extremities), laboratory tests (elevated cardiac enzymes, lactate, myoglobin, or creatine kinase), and impaired blood flow to the corresponding arteries on radiographic findings [computed tomography angiography (CTA) scan or ultrasound] (Supplementary Material 1). The exclusion criteria included relapse, declined treatment, a lack of data required to calculate inﬂammatory and nutritional indices, or loss to follow-up. The workflow of this study is shown in Figure 1.

#### Predictors and endpoint

A total of 19 variables, including age, sex, BMI, the inflammatory index (IMI), the nutritional index (NI), inflammatory and nutritional laboratory values (IMNLV), and inflammatory and nutritional indices (IMNI), were collected. The inflammatory and nutritional indices were calculated as described in Supplementary Material 2. IMNLV, IMNI, IMI and NI were separately used as predictors along with age, sex, and BMI to develop the models. The variables are presented as continuous variables, except for sex, which is represented as a binary variable. The primary endpoint was 1-year mortality after the diagnosis of ATAAD with malperfusion.

### Data analysis

#### Data preprocessing

Data analysis was carried out for 1-year mortality. The full dataset was divided into model development (70%) and model testing (30%) datasets. The model used stratified random sampling and resulted in approximately equal frequencies of outcomes in both subsets.

#### Model development and testing

The dataset was used to establish four predictive models for binary classification of the modeled outcome: a logistic regression model, an XGBoost model, a random forest model and a deep neural network. Candidate variable selection for all the models involved all the predictors in the dataset. The models were trained with a stratified 5-fold cross-validation method, in which the development dataset was partitioned into five data “folds”. To improve training, we rebalanced the training subset with respect to the outcome via synthetic minority oversampling (SMOTE). With the rebalanced training subset, hyperparameter tuning was performed by applying a random grid search strategy to select an optimal hyperparameter set. This cross-validation was repeated five times so that each fold was used for validation exactly once. The receiver operating characteristic (ROC) area under the curve (AUC) was calculated for each validation fold, and the optimal threshold was determined by minimizing the distance from the AUC to the (0, 1) point.

Once cross-validation was completed, the model with the highest validation AUC was selected, and its corresponding hyperparameters were saved. This model was then evaluated on a holdout testing dataset. For the testing dataset, we computed the ROC AUC and applied the optimal prediction threshold determined during validation to predict outcomes. We also assessed the models' performances by calculating key metrics such as the sensitivity, specificity, positive predictive value (PPV), negative predictive value (NPV), each with their respective 95% confidence intervals. To evaluate clinical utility, decision curve analysis (DCA) was used to quantify the net benefit of each model across a range of probability thresholds. Additionally, calibration curves were generated on the internal test set, and brier scores were calculated to quantify the overall accuracy of predicted probabilities.

#### Model interpretability

To interpret how individual features contribute to the model's ability to predict 1-year mortality, we computed feature importance values via both model-specific and *post hoc* explainability methods. For model-specific feature importance, we extracted feature importance scores from the ensemble model by analyzing their respective decision trees. Each feature's importance was determined on the basis of its role in tree node splits, quantified by metrics such as information gain and Gini index reduction. The cumulative importance values across all trees were normalized for individual features to indicate their relative influence on the model's predictions.

SHAPley additive explanations (SHAP) is a *post hoc* explainability method designed as an additive feature attribution method; it represents the contribution of each feature to the ML model prediction by constructing an interpretable approximation of the original model. *Post hoc* explainability is required to understand complex models, such as XGBoost and random forest, that forgo simplicity and explainability for increased model performance. SHAP values are a unified measure of 4 feature attribution models that quantitatively decide how important a feature is to the decision-making process of the model. Additionally, we performed decision curve analysis, a method that accounts for the different weights of misclassification types, providing a direct clinical interpretation. The net benefit of each model was calculated and graphically displayed across a range of threshold probabilities, illustrating the clinical utility of each model.

### Statistical analysis

For comparisons between the survivor and non-survivor groups, continuous variables were tested for normality using the Shapiro–Wilk test. Normally distributed variables were expressed as mean ± standard deviation (SD) and compared between groups using Student's *t*-test. Non-normally distributed variables were presented as median (interquartile range, IQR) and compared using the Mann–Whitney *U*-test. Categorical variables were summarized as counts (percentages) and compared between groups using the Chi-square test. The classification performance was assessed via the ROC AUC, sensitivity, specificity, PPV, and NPV metrics. Bootstrapping was used to estimate the variability in all the metrics, and 95% confidence intervals were calculated. Pairwise DeLong tests were conducted to statistically compare ROC AUC performance across individual models. Statistical significance was taken as *P* < 0.05.

### Software and packages

Python version 3.12.6 was used to perform all analyses. The scikit-learn (version 1.5.2) package was used for data preprocessing, model development, and calculation of performance metrics. Logistic regression, random forest, and deep neural networks were implemented in scikit-learn (version 1.5.2), SMOTE-Tomek was implemented in imblearn (version 0.12.3), and the xgboost model was implemented in the xgboost (version 2.1.1) package. Statistical testing was performed via scipy (version 1.14.1).

## Results

Of the 443 consecutive patients screened, 10 were excluded, and the follow-up completeness was 97.74%. Table 1 summarizes the baseline characteristics of included 433 patients stratified by 1-year survival status. Of the cohort, 352 (81.3%) were survivors and 81 (18.7%) were non-survivors. The median age was 48 years (IQR: 40–57) overall, 47 years (40–57) in survivors, and 51 years (42–58) in non-survivors (*P* = 0.06). Males comprised 65.1% of the cohort, with a similar distribution between survivors (65.1%) and non-survivors (65.4%) (*P* = 0.95). The median BMI was 21 (IQR: 18–25) with no significant group difference (*P* = 0.18). Most inflammatory and nutritional markers, including C-reactive protein, white blood cell count, and lymphocyte count, did not differ significantly between groups; however, platelet count was significantly lower in non-survivors (*P* = 0.02). Prognostic nutritional index was also lower (*P* < 0.01) and instant nutritional assessment score higher (*P* = 0.02) in non-survivors. Coronary malperfusion was more common in non-survivors (58.0% vs. 5.4%, *P* < 0.01). Non-survivors also had a higher prevalence of aortic atherosclerosis (9.9% vs. 4.0%, *P* = 0.04) and a longer median time from symptom onset to surgery [7 (IQR: 4–10) vs. 4 (IQR: 2–6) hours, *P* < 0.01] (Supplementary Table S1).

**Table 1:** Baseline characteristics of patients.

Variables	Overall (*n* = 433)	Survivors (*n* = 352)	Non-survivors (*n* = 81)	*P*-value
Age, Median (IQR)	48 (40–57)	47 (40–57)	51 (42–58)	0.06
Gender, *n* (%)				
Male	282 (65.13)	229 (65.06)	53 (65.43)	0.95
Female	151 (34.87)	123 (34.94)	28 (34.58)	
Body mass index, Median (IQR)	21 (18–25)	21 (18–24)	22 (18–26)	0.18
Albumin, Median (IQR)	3.84 (3.76–3.95)	3.85 (3.77–3.95)	3.77 (3.61–4.02)	0
C-reactive protein, Median (IQR)	2.84 (2–3.66)	2.81 (1.95–3.59)	2.90 (2.15–3.83)	0.29
Platelet count, Median (IQR)	164.42 (152.14–177.92)	165.37 (153.16–179.20)	159.69 (146.76–172.51)	0.02
Neutrophil count, Median (IQR)	5.02 (4.16–6.17)	5.01 (4.12–6.07)	5.14 (4.28–6.60)	0.15
Monocyte count, Median (IQR)	0.66 (0.50–0.81)	0.67 (0.50–0.84)	0.63 (0.48–0.75)	0.13
Lymphocyte count, Median (IQR)	1.93 (1.40–2.45)	1.90 (1.39–2.45)	1.95 (1.46–2.43)	0.62
Total cholesterol, mean (SD)	155.79 (13.31)	155.95 (13.53)	155.12 (12.40)	0.60
White blood cell count, Median (IQR)	7.96 (6.84–9.25)	7.92 (6.84–9.14)	8.18 (6.92–9.46)	0.22
Neutrophil to lymphocyte ratio, Median (IQR)	2.76 (2.02–3.61)	2.77 (1.98–3.61)	2.72 (2.16–3.54)	0.73
Monocyte to lymphocyte ratio, Median (IQR)	0.34 (0.25–0.48)	0.35 (0.25–0.49)	0.30 (0.23–0.44)	0.21
Platelet to lymphocyte ratio, Median (IQR)	86.05 (65.94–117.70)	87.75 (67.40–119.31)	84.27 (62.21–114.28)	0.30
Systemic immune inflammation index, Median (IQR)	442.04 (328.32–602.23)	443.45 (327.12–603.61)	421.77 (347.22–588.59)	0.83
Systemic inflammation response index, Median (IQR)	1.72 (1.13–2.50)	1.76 (1.14–2.56)	1.56 (1.08–2.38)	0.50
Controlling nutritional status score, Median (IQR)	1 (1–2)	1 (1–2)	1 (1–3)	0.18
Prognostic nutritional index, Median (IQR)	38.41 (37.61–39.51)	38.51 (37.71–39.51)	37.71 (36.11–40.21)	<0.01
Instant nutritional assessment, Median (IQR)	1 (1–2)	1 (1–2)	1 (1–2)	0.02
Malperfusion, *n* (%)				
Coronary	66 (15.24)	19 (5.40)	47 (58.02)	<0.01
Cerebral	136 (31.41)	93 (26.42)	43 (53.09)	
Spinal cord	9 (2.08)	8 (2.27)	1 (1.23)	
Mesenteric	106 (24.48)	87 (24.72)	19 (23.46)	
Renal	103 (23.78)	91 (25.85)	12 (14.81)	
Lower leg	180 (41.57)	160 (45.45)	20 (24.69)	
Dissection site, *n* (%)				
Above renal arteries	0 (0)	0 (0)	0 (0)	1
Below renal arteries	433 (100)	352 (100)	81 (100)	

The performance of four machine learning models (a logistic regression model, an XGBoost model, a random forest model and a deep neural network) in the prediction of 1-year mortality was assessed via different feature sets: IMNLV, IMNI, IMI and NI. Key metrics, including the ROC AUC, sensitivity, specificity, PPV, and NPV, were used to evaluate the models' effectiveness (Table 2).

**Table 2:** Prediction ability of the 4 machine learning models in 1-year mortality of acute type A aortic dissection with malperfusion.

Datasets	Model	Best threshold	AUC (95% CI)	Sensitivity (95% CI)	Specificity (95% CI)	PPV (95% CI)	NPV (95% CI)
Inflammatory and nutritional laboratory values	Logistic regression	0.72	0.5695 (0.4186–0.7084)	0.2836 (0.1176–0.4828)	0.9425 (0.8928–0.9810)	0.5247 (0.2500–0.8000)	0.8545 (0.7899–0.9138)
	XGBoost	0.76	0.7901 (0.6738–0.8857)	0.4948 (0.2857–0.7001)	0.9432 (0.8956–0.9818)	0.6608 (0.4375–0.8697)	0.8930 (0.8291–0.9460)
	Random forest	0.43	0.8225 (0.7184–0.9136)	0.7082 (0.5238–0.8750)	0.8107 (0.7315–0.8807)	0.4581 (0.3000–0.6216)	0.9249 (0.8617–0.9707)
	Deep neural network	0.61	0.6422 (0.4927–0.7887)	0.3790 (0.1923–0.6000)	0.9440 (0.8990–0.9822)	0.6096 (0.3333–0.8750)	0.8683 (0.8053–0.9279)
Inflammatory and nutritional indexes	Logistic regression	0.76	0.6780 (0.5458–0.8160)	0.2886 (0.1111–0.5000)	0.9905 (0.9700–1.0000)	0.8707 (0.5710–1.0000)	0.8616 (0.8000–0.9187)
	XGBoost	0.29	0.7334 (0.6115–0.8488)	0.6688 (0.4737–0.8519)	0.7164 (0.6336–0.7947)	0.3466 (0.2041–0.4884)	0.9059 (0.8434–0.9634)
	Random forest	0.48	0.7075 (0.5604–0.8317)	0.5032 (0.3041–0.7037)	0.8865 (0.8241–0.9439)	0.5000 (0.2915–0.7201)	0.8879 (0.8218–0.9434)
	Deep neural network	0.62	0.6285 (0.4932–0.7570)	0.2915 (0.1303–0.4832)	0.9626 (0.9238–0.9910)	0.6407 (0.3333–0.9170)	0.8562 (0.7949–0.9145)
Nutritional index	Logistic regression	0.56	0.6299 (0.4721–0.7786)	0.3729 (0.1739–0.5667)	0.9529 (0.9065–0.9904)	0.6432 (0.3633–0.8889)	0.8701 (0.8091–0.9244)
	XGBoost	0.68	0.7358 (0.6158–0.8515)	0.4949 (0.2857–0.6897)	0.9439 (0.8972–0.9813)	0.6637 (0.4286–0.8750)	0.8932 (0.8349–0.9402)
	Random forest	0.62	0.7169 (0.5636–0.8481)	0.4600 (0.2590–0.6670)	0.9628 (0.9223–0.9910)	0.7333 (0.5000–0.9333)	0.8881 (0.8250–0.9455)
	Deep neural network	0.63	0.7036 (0.5594–0.8407)	0.4145 (0.2174–0.6297)	0.9810 (0.9528–1.0000)	0.8280 (0.5556–1.0000)	0.8818 (0.8235–0.9381)
Inflammatory index	Logistic regression	0.63	0.6407 (0.5091–0.7657)	0.3809 (0.1904–0.5714)	0.8793 (0.8108–0.9386)	0.4147 (0.2105–0.6364)	0.8637 (0.7927–0.9266)
	XGBoost	0.4	0.5539 (0.4250–0.6727)	0.4181 (0.2272–0.6000)	0.7077 (0.6226–0.7885)	0.2430 (0.1212–0.3714)	0.8441 (0.7683–0.9157)
	Random forest	0.24	0.5363 (0.4013–0.6595)	0.8765 (0.7273–1.0000)	0.2264 (0.1538–0.3093)	0.2041 (0.1321–0.2788)	0.8903 (0.7500–1.0000)
	Deep neural network	0.5	0.4981 (0.3697–0.6214)	0.3315 (0.1429–0.5263)	0.7526 (0.6698–0.8302)	0.2337 (0.0968–0.3793)	0.8317 (0.7586–0.9043)

**Figure 2:**
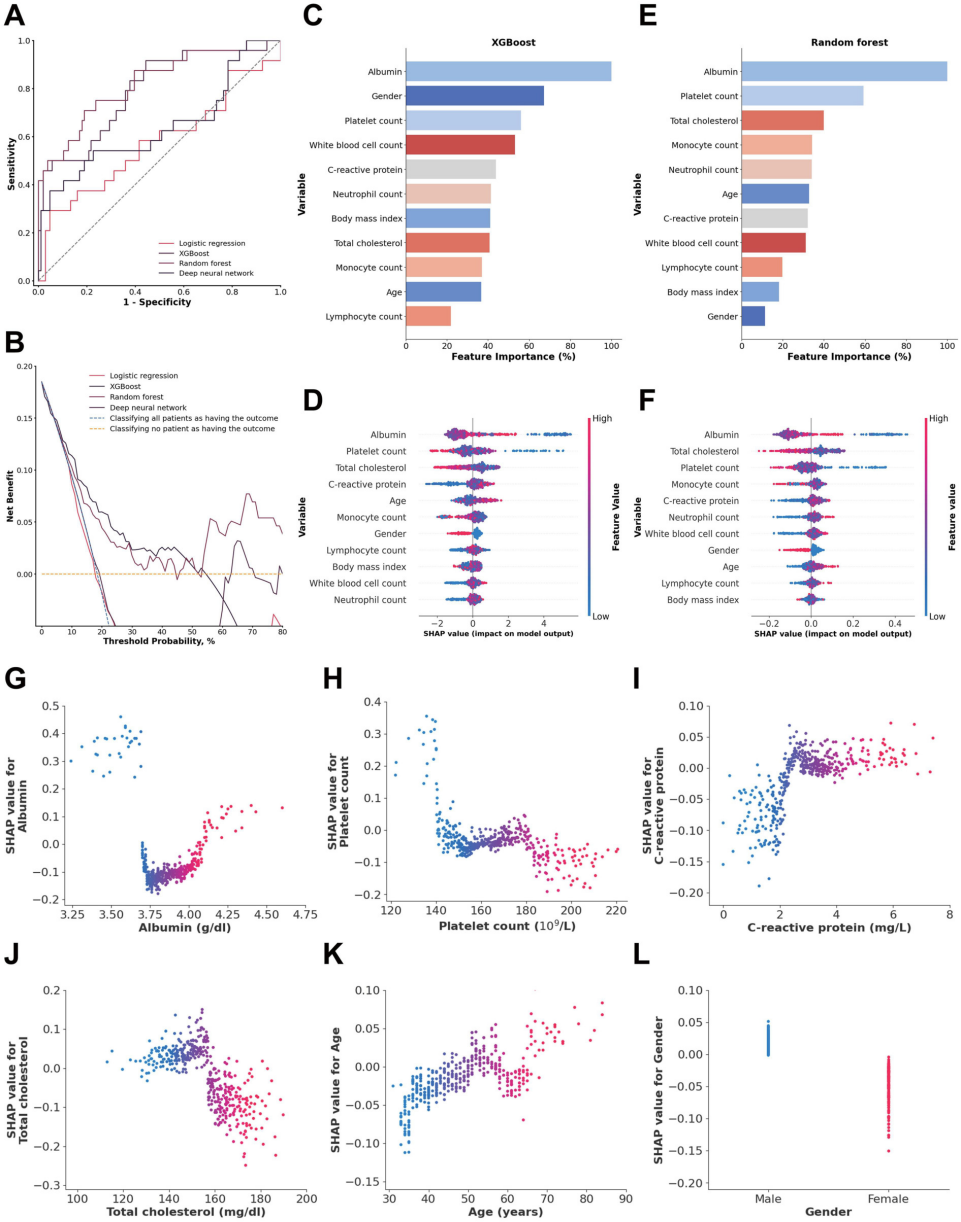
**(A)** ROC analysis results of the four models, including logistic regression, random forest, deep neural network, and XGBoost. **(B)** Decision curve analysis illustrating the potential clinical application of the prediction models (logistic regression, random forest, deep neural network, and XGBoost) across a range of threshold probabilities. **(C)** Importance matrix of the XGBoost model. **(D)** SHAP summary plot of all the features in the XGBoost model. **(E)** Importance matrix of the random forest model. **(F)** SHAP summary plots of all features in the random forest model. **(G–L)** Beeswarm plot illustrating the SHAP values for albumin, platelet count, total cholesterol, C-reactive protein, age and gender in the random forest model.

Inflammatory and nutritional laboratory values. The random forest model demonstrated the highest performance, with an AUC of 0.8225 (95% CI: 0.7184–0.9136), achieving a sensitivity of 0.7082 (95% CI: 0.5238–0.8750) and a specificity of 0.8107 (95% CI: 0.7315–0.8807). The XGBoost model showed a slightly lower AUC of 0.7901 (95% CI: 0.6738–0.8857), with lower sensitivity (0.4948, 95% CI: 0.2857–0.7001) but higher specificity (0.9432, 95% CI: 0.8956–0.9818) (Figure 2A). DeLong tests confirmed random forest model was superior to all other models (all *p* < 0.01). The decision curve analysis revealed that the random forest model demonstrated the most significant net benefit across a wide range of 10%–50% probabilities in predicting outcomes (Figure 2B). The calibration curve further showed that the random forest model was well calibrated (Brier score = 0.1288) (Supplementary Figure S1), indicating its reliability in predicting 1-year mortality for type A aortic dissection with malperfusion.

The feature importance and SHAP value of variables in the XGBoost and random forest models for each data source are shown in Figures 2C–. SHAP values provide a nuanced view of how each feature impacts individual predictions, capturing variability and localized interactions. By comparing feature importance of the XGBoost and random forest models, it was clear that albumin, platelet count, C-reactive protein and total cholesterol overlapped among the 5 most important features of both models. In addition, albumin was also identified as a shared top 5 feature in both models based on SHAP values, beyond platelet count.

An albumin concentration <3.7 g/dl and >4.09 g/dl, a platelet count <144.2 × 10^9^/L, a total cholesterol concentration <157.97 mg/dl, and a C-reactive protein concentration >2.32 mg/dl were associated with an increase in model SHAP output, reflecting a model-detected trend toward increased mortality risk. Also, age >65 years and male sex were associated with an increase in the model output linked to a higher predicted risk of 1-year mortality (Figures 2G–L).

### Inflammatory and nutritional indices

**Figure 3:**
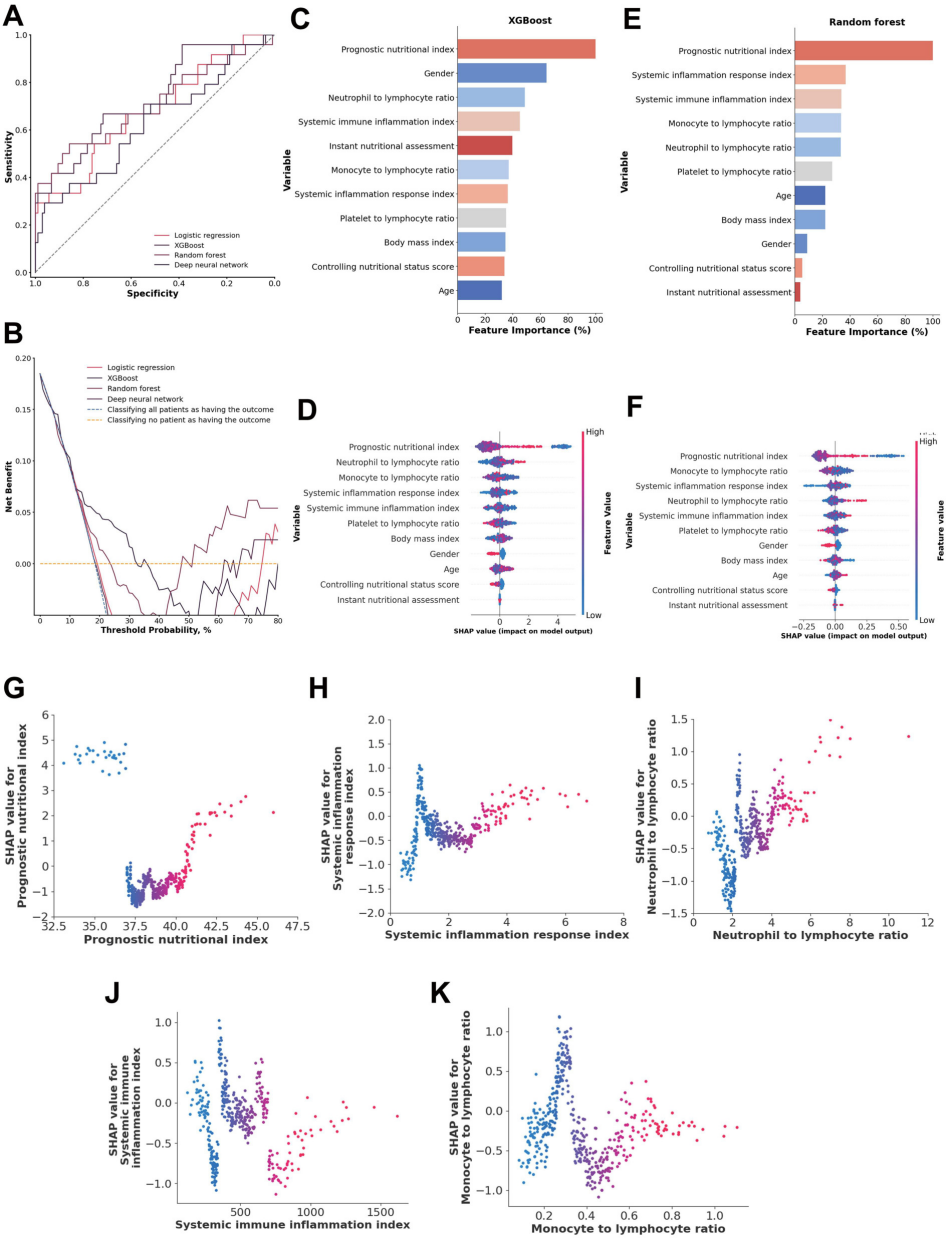
**(A)** ROC analysis results of the four models, including logistic regression, random forest, deep neural network, and XGBoost. **(B)** Decision curve analysis illustrating the potential clinical application of the prediction models (logistic regression, random forest, deep neural network, and XGBoost) across a range of threshold probabilities. **(C)** Importance matrix plot of the XGBoost model. **(D)** SHAP summary plots of all the features in the XGBoost model. **(E)** Importance matrix plot of the random forest model. **(F)** SHAP summary plots of all features in the random forest model. **(G–K)** Beeswarm plot illustrating the SHAP values for the systemic inflammation response index, neutrophil-to-lymphocyte ratio, monocyte-to-lymphocyte ratio, prognostic nutritional index (PNI) and systemic immune inflammation index features in the XGBoost model.

When the inflammatory and nutritional indexes were used, the XGBoost model achieved the highest performance, with an AUC of 0.7334 (95% CI: 0.6115–0.8488), a sensitivity of 0.6688 (95% CI: 0.4737–0.8519), and a specificity of 0.7164 (95% CI: 0.6336–0.7947). The random forest model had a slightly lower AUC of 0.7075 (95% CI: 0.5604–0.8317), with a sensitivity of 0.5032 (95% CI: 0.3041–0.7037) and a higher specificity of 0.8865 (95% CI: 0.8241–0.9439) (Figure 3A). DeLong tests confirmed the XGBoost model was superior to all other models (all *p* < 0.01). The XGBoost model performed exceptionally well when the threshold probabilities were between 0.12 and 0.34 (Figure 3B). Moreover, the XGBoost model also demonstrated good calibration (Brier score = 0.1506) (Supplementary Figure S1), indicating its reliability in predicting the 1-year mortality of acute type A aortic dissection with malperfusion.

A comparison of the feature importance of the inflammatory and nutritional indices in the XGBoost and random forest models revealed that the prognostic nutritional index, systemic immune inflammation index, and neutrophil-to-lymphocyte ratio were shared among the top 5 most important features in both models. Additionally, SHAP values indicated that these three indices were consistently shared among the top 5 features in both models, along with the monocyte-to-lymphocyte ratios and systemic inflammation response index (Figures 3C–F).

A systemic inflammation response index from 1.26 to 1.62 and >3.96, a neutrophil-to-lymphocyte ratio >4.01, a monocyte-to-lymphocyte ratio from 0.24 to 0.23, and a prognostic nutritional index <37.22 and >40.81 were associated with higher SHAP values, indicating a model-predicted trend toward increased 1-year mortality risk. The systemic immune inflammation index is more scattered approximately 0, suggesting that those indices are both positive and negative in different patients (Figures 3G–K).

### Nutritional Index

**Figure 4:**
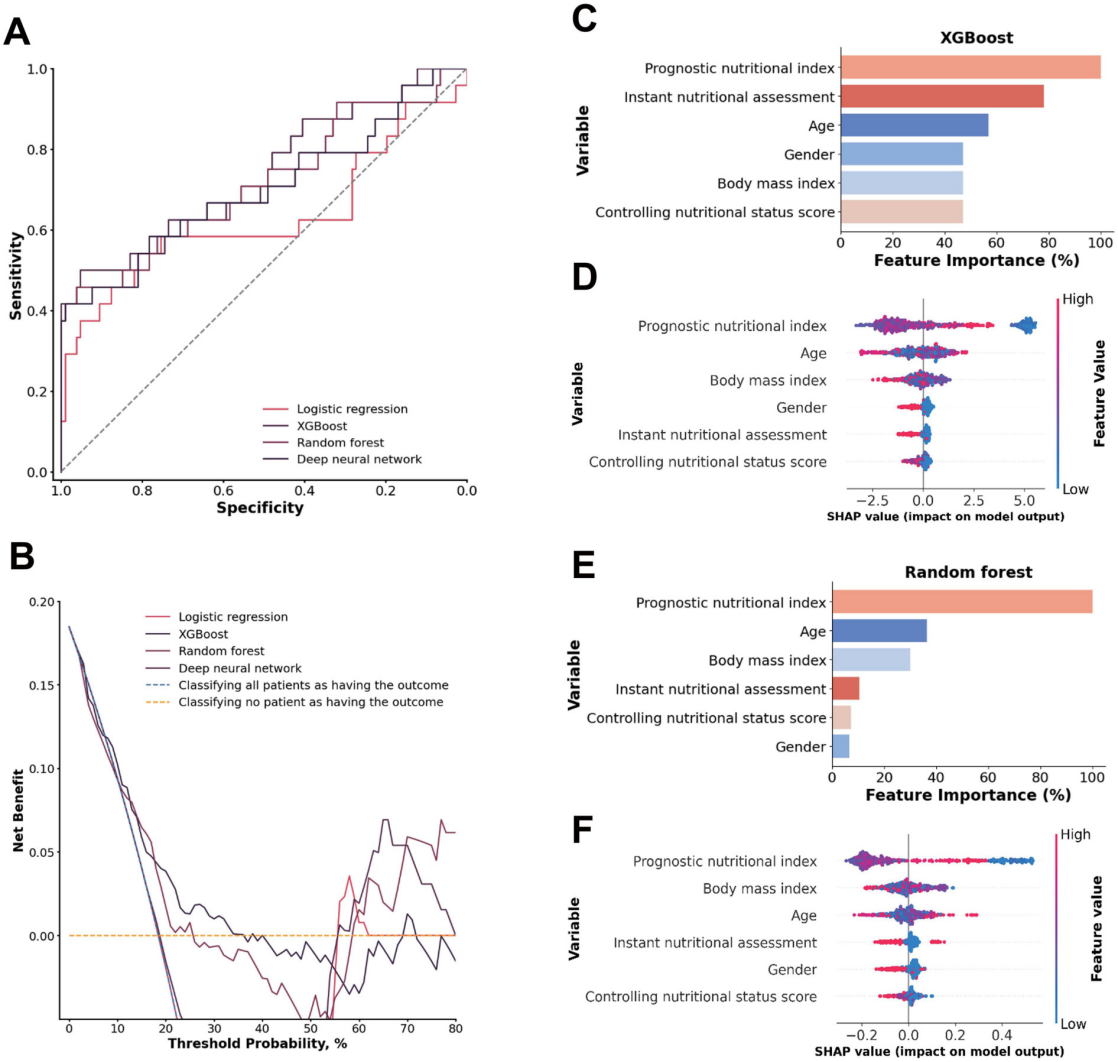
**(A)** ROC analysis results of the four models, including logistic regression, random forest, deep neural network, and XGBoost. **(B)** Decision curve analysis illustrating the potential clinical application of the prediction models (logistic regression, random forest, deep neural network, and XGBoost) across a range of threshold probabilities. **(C)** Importance matrix plot of the XGBoost model. **(D)** SHAP summary plots of all the features in the XGBoost model. **(E)** Importance matrix plot of the random forest model. **(F)** SHAP summary plots of all features in the random forest model.

For the nutritional index, the XGBoost model achieved the highest AUC at 0.7358 (95% CI: 0.6158–0.8515), with a sensitivity of 0.4949 (95% CI: 0.2857–0.6897) and a specificity of 0.9439 (95% CI: 0.8972–0.9813). The random forest model showed a comparable AUC of 0.7169 (95% CI: 0.5636–0.8481), with slightly lower sensitivity of 0.4600 (95% CI: 0.2590–0.6670) but higher specificity of 0.9628 (95% CI: 0.9223–0.9910). DeLong tests confirmed the XGBoost model was superior to all other models (all *p* < 0.01). The XGBoost model performed particularly well when the threshold probability was between 0.13 and 0.35, highlighting its reliability for evaluating patients with malperfusion and ATAAD to predict 1-year mortality (Figure 4B).

A comparison of the feature importance of the nutritional indices in the XGBoost and random forest models revealed that the prognostic nutritional index was a shared feature from the top 3 most important features in both models. Furthermore, the top 3 features according to the SHAP value were the prognostic nutritional index, age and BMI in both models. The prognostic nutritional index exhibited the same pattern as previously described, suggesting that an abnormal nutritional status contributes significantly to an increased model-predicted trend for1-year mortality risk. Age and BMI are more scattered approximately 0, suggesting that those indices are both positive and negative in different patients (Figures 4C–F).

### Inflammatory indices

**Figure 5:**
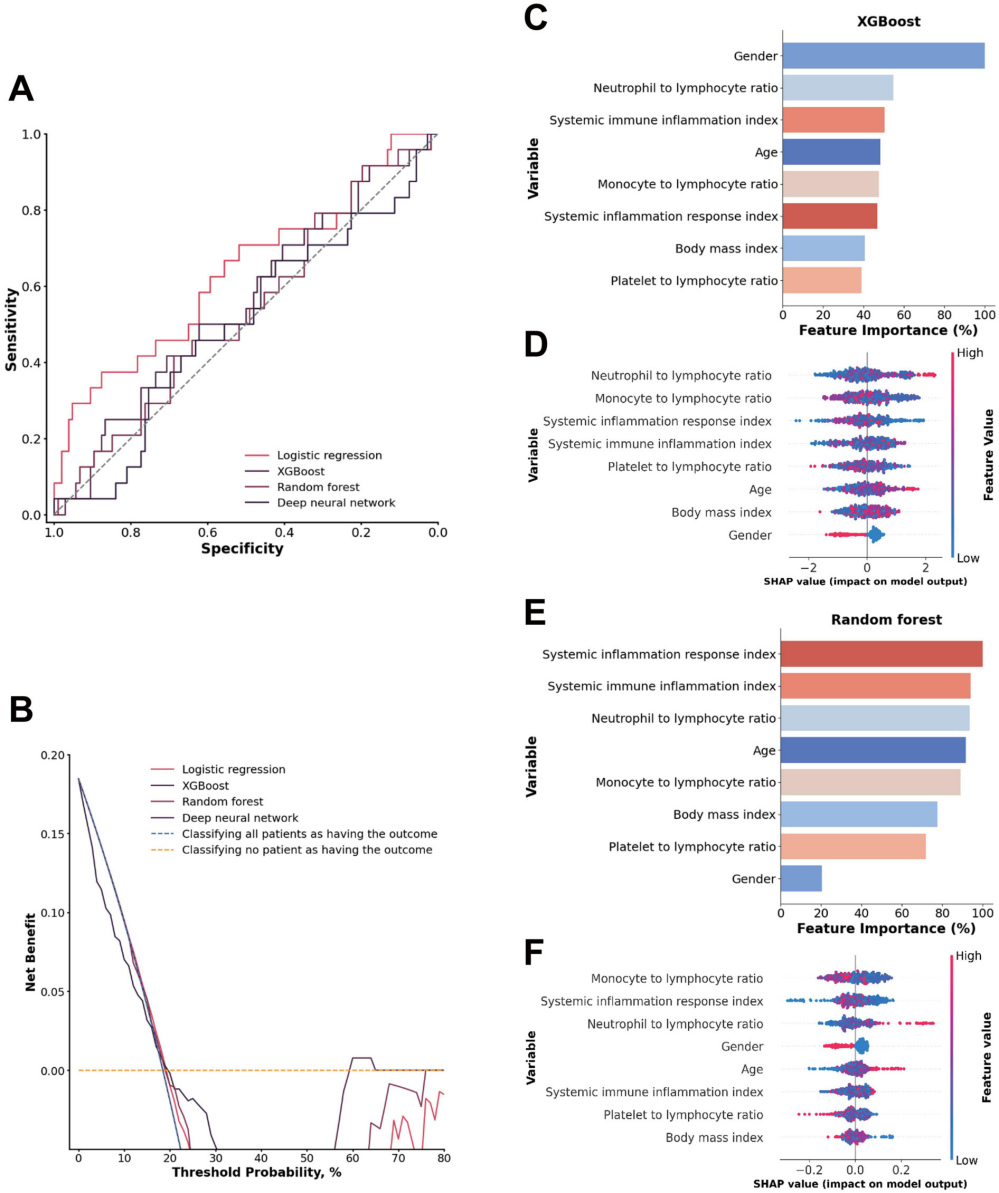
**(A)** ROC analysis results of the four models, including logistic regression, random forest, deep neural network, and XGBoost. **(B)** Decision curve analysis illustrating the potential clinical application of the prediction models (logistic regression, random forest, deep neural network, and XGBoost) across a range of threshold probabilities. **(C)** Importance matrix plot of the XGBoost model. **(D)** SHAP summary plots of all the features in the XGBoost model. **(E)** Importance matrix plot of the random forest model. **(F)** SHAP summary plots of all features in the random forest model.

Among the four models evaluated for predicting the inflammatory response in ATAAD patients with malperfusion, all demonstrated limited ability to distinguish between classes, with moderate sensitivity and specificity values that reflect a weaker overall discriminatory power for this application (Figures 5A,B).

A comparison of the feature importance of the inflammatory indices in the XGBoost and random forest models revealed that only the systemic immune inflammation index was shared by the top 3 most important features in both models. Furthermore, in both models, the top 3 features in terms of the SHAP value were the systemic inflammation response index, monocyte-to-lymphocyte ratio, and neutrophil-to-lymphocyte ratio. These inflammation indicators may have limited predictive power in their specific combination, which could contribute to the model's overall limited performance (Figures 5C–F).

**Figure 6:**
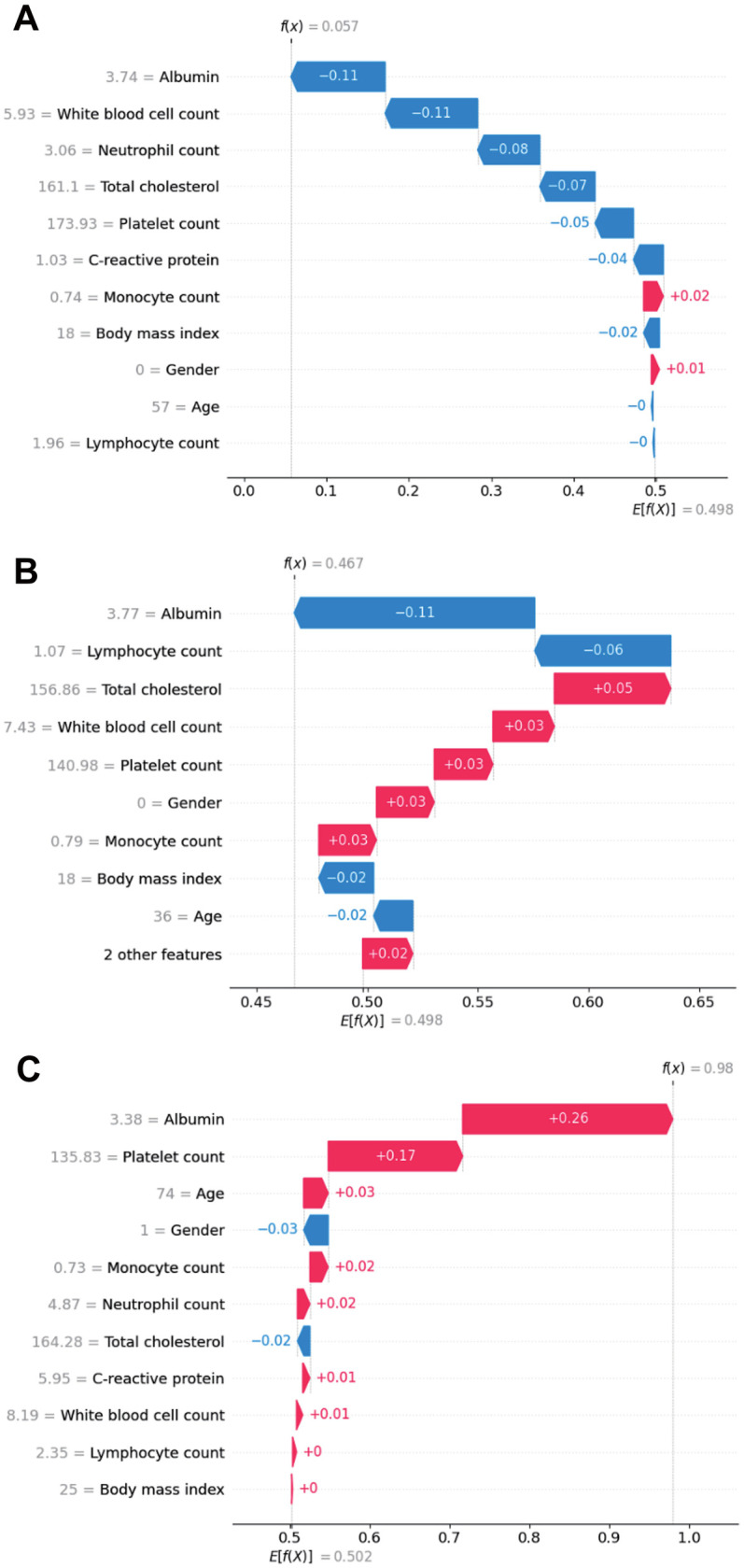
SHAP waterfall plot illustrating the contribution of individual features, including inflammatory and nutritional laboratory values, age, BMI and sex, to a specific predictions generated by the random forest model in real patients. **(A)** Patient 1; **(B)** Patient 2; **(C)** Patient 3.

Individual patients included in the random forest model. SHAP value visualizations for three individual patients in a waterfall plot of the random forest model illustrate the impact of each variable on model predictions for the inflammatory and nutritional laboratory values. As shown in Figure 6A, the level of albumin, white blood cell count, and neutrophil count had strong negative impacts, decreasing the predicted outcome. Conversely, the monocyte count and body mass index had minor positive effects. Figure 6B shows a similar trend in which the ALB concentration and lymphocyte count are influential negative predictors, whereas sex and monocyte count slightly increase the prediction. Figure 6C shows that Albumin has a notably high positive impact, indicating its significant contribution to an elevated prediction score, alongside a smaller contribution from the Platelet Count. Overall, these visualizations highlight Albumin as a consistently impactful feature, with its effect varying between negative and positive contributions, depending on the patient. This variation underscores the complexity of feature interactions within the model and their individualized impact on prediction scores (Figures 6A–C).

## Discussion

In this retrospective study, we developed a predictive machine learning model for evaluating ATAAD with a 1-year mortality rate incorporating nutritional and inflammatory factors. Among these factors, albumin, platelet count, total cholesterol, and c-reactive protein had the highest predictive value for mortality among the nutritional and inflammatory factors. The predictive values of these factors indicate that these factors are the most reliable predictors of 1-year mortality in ATAAD patients with malperfusion. The random forest model had excellent prediction ability: it had an AUC of 0.8225 and good discrimination and calibration power in predicting 1-year mortality in the inflammatory and nutritional laboratory values dataset.

Traditional risk models tend to focus on demographic and clinical factors, such as age, sex, and disease history. However, emerging biomarkers related to inflammation and nutrition play significant roles in the pathophysiology of AAD (15–17). Previous studies have demonstrated that inflammatory factors and nutritional factors can significantly influence patient prognosis (13, 18). The importance of inflammatory and nutritional factors in predicting patient outcomes is well documented in the cardiovascular disease literature, but few studies have systematically integrated these parameters into ML models for ATAAD patients with perfusion.

Nutrition has been closely linked with AAD mortality rates, especially since malnutrition has been associated with exacerbations of cardiovascular disorders (19). Predictors such as albumin levels, the prognostic nutritional index, and other tests can reflect a patient's nutritional status as well as the severity of their condition (20–22). Nutritional factors such as albumin levels and the prognostic nutritional index (which reflect a patient's overall health and recovery capacity) are similarly critical for predicting outcomes (23, 24). For example, a patient's prognostic nutritional index has been linked to surgical outcomes and long-term mortality under cardiovascular conditions and has been shown to be an accurate indicator of their future recovery (25, 26). Malnutrition has been linked to poorer outcomes in various cardiovascular conditions, and it can exacerbate the course of diseases such as AAD by weakening the body's ability to recover from acute events (26).

Similarly, inflammation plays a pivotal role in the degradation of the aortic wall, leading to complications such as aortic rupture or malperfusion (27–29). Elevated inflammatory markers such as neutrophils, CRP, and fibrinogen are often indicative of heightened immune responses, which can accelerate tissue damage and worsen prognosis. Increased inflammation is often a sign of increased protease and ROS production, which may lead to increased smooth muscle apoptosis and worsening of conditions such as AAD (30–34). When used in combination, both inflammatory and nutritional factors can create a holistic understanding of a patient's state of health, which can support model-based predictions of mortality in conditions such as AAD.

The performance of the ML models, especially XGBoost and random forest, was promising. These models achieved AUCs around 0.80 when inflammatory and nutritional laboratory values were used as predictors, such as albumin, total cholesterol, and various inflammatory factors, such as white blood cell count, monocyte, neutrophil, and lymphocyte count, significantly improving the predictive performance compared with traditional models that exclude these factors. In particular, the random forest model demonstrated excellent performance, with an AUC of 0.8225, a sensitivity of 0.7082, and a specificity of 0.8107, making it suitable for identifying high-risk patients who may require more aggressive monitoring and intervention.

The inclusion of inflammatory and nutritional indices also led to good model performance, although it was slightly lower than when raw laboratory values were used. This suggests that while indices provide a useful summary of a patient's condition, the granular data captured by individual laboratory values offer more predictive power. These findings highlight the complexity of AAD pathophysiology and the need for detailed biochemical data to accurately predict outcomes. Importantly, the machine learning models were less accurate in their predictions when nutritional and inflammatory factors were considered separately. The parameters of the index model consist of a controlling nutritional index, a prognostic nutritional index, and different inflammatory indices, utilizing evaluations of neutrophils, platelets, monocytes, and lymphocytes, as they are recognized as crucial mediators of inflammatory responses.

In clinical practice, these ML models could aid early-stage AAD management by identifying patients at higher risk for 1-year mortality, enabling more intensive monitoring and personalized interventions. SHAP analysis revealed nonlinear relationships, including a U-shaped association between serum albumin and mortality risk, reflecting complex, non-monotonic effects and potential feature interactions rather than fixed thresholds. Clinically, low albumin indicates poor nutritional status, systemic inflammation, and adverse surgical prognosis (35, 36), while markedly elevated levels may signal hemoconcentration from hypovolemia or shock (37). The patients with abnormal albumin could be flagged for measures to mitigate malperfusion or control inflammation.

In our cohort, over half of the non-survivors had coronary (58.0%) and/or cerebral malperfusion (53.1%) and often died from acute myocardial infarction, stroke, or fatal mechanical complications before ischemia or inflammatory markers such as C-reactive protein and troponin I rose to clinically significant levels, explaining the relatively low CRP and troponin I in both groups (Supplementary Table S1). For such patients, mortality may be driven more by mechanical and hemodynamic collapse than by systemic derangements. These observations underscore the importance of integrating clinical and hemodynamic parameters—such as ischemia time, presence of tamponade, and brachiocephalic artery occlusion—alongside laboratory predictors in future prognostic models to better capture the multifactorial nature of mortality risk in ATAAD with malperfusion.

The decision curve analysis (DCA) data further emphasize the promising potential of machine learning models in clinical applications. The random forest, in particular, demonstrated the highest net benefit across a range of threshold probabilities, indicating its superior performance in balancing the risks of false positives and false negatives. This is especially relevant in a clinical setting where over- and undertriage can have significant consequences. Over-triaging may lead to unnecessary interventions and increased healthcare costs, whereas undertriaging could result in delayed treatment and increased mortality. The ability of the random forest model to offer the best trade-off between these two extremes makes it a valuable tool in the clinical management of AAD patients with malperfusion through the use of inflammatory and nutritional laboratory values.

## Limitations and future directions

While the findings of this study are promising, several limitations should be considered. (1) The sample size of 433 patients, although sufficient for initial model development, is relatively small for robust ML applications, particularly when complex algorithms such as random forest and XGBoost are used. (2) Additionally, the study was conducted at a single center, which limits the generalizability of the findings. Larger, multicenter studies are needed to validate the model's performance in diverse patient populations. (3) Another limitation is the retrospective nature of the study, which introduces potential biases related to data collection and patient selection. Prospective studies would provide more robust evidence and allow for real-time application of ML models in clinical practice. (4) Moreover, the study did not explore the mechanistic pathways linking nutritional and inflammatory factors to ATAAD with perfusion outcomes in detail. Future research should aim to uncover the biological mechanisms through which these factors influence disease progression. Future models could also integrate quantitative malperfusion burden scores, such as the number and extent of vascular territories involved, to potentially improve predictive performance.

## Conclusion

This study highlights the potential of machine learning models that incorporate nutritional and inflammatory factors to evaluate patients with malperfusion and acute type A aortic dissection to predict 1-year mortality. The results suggest that these factors play a significant role in patient outcomes and should be considered in risk stratification models. While the XGBoost and random forest models showed high predictive accuracy, further research is needed to validate these findings in larger, multicenter studies. Additionally, integrating these models into clinical practice could improve patient outcomes by enabling more personalized and timely interventions. By emphasizing the importance of nutritional and inflammatory factors, this study paves the way for more comprehensive and individualized treatment approaches for AAD, ultimately aiming to better inform physicians, reduce mortality and improve the quality of life of affected patients.

## Data Availability

The original contributions presented in the study are included in the article/Supplementary Material, further inquiries can be directed to the corresponding author.

## Supplementary material

Supplementary Figure S1. Calibration curves for **(A)** the Random Forest model using inflammatory and nutritional laboratory values and **(B)** the XGBoost model using inflammatory and nutritional indices. The dashed line (Ideal) represents perfect calibration, where predicted probabilities exactly match observed outcomes. The blue solid line (Apparent) shows the observed performance of the model, while the yellow solid line (Bias-corrected) reflects bias-adjusted performance.
