# Supplementary material for: Machine learning-based prediction of 1-year mortality using nutritional and inflammatory factors for type A acute aortic dissection with malperfusion
Source: Front Cardiovasc Med. 2025 Sep 29;12:1539267. doi: 10.3389/fcvm.2025.1539267 (PMC12515875; doi:10.3389/fcvm.2025.1539267)
Supplement: Supplementary file 1 [file Datasheet1.pdf]

**Supplementary material 1**

Preoperative findings were used to classify the following malperfusion:

1. Coma is reported as a coma or coma/altered consciousness on admission, defined as complete or partial mental unresponsiveness (beyond that expected from anesthesia) or absence of psychological or physiologically appropriate responses to stimulation.

2. Coronary malperfusion is defined as the presence of myocardial ischemia, myocardial infarction, or electrocardiogram findings of new Q waves or ST-segment elevations.

3. Limb ischemia includes patients with reported ischemic peripheral neuropathy and ischemic lower extremities.

4. Neurological deficit includes those with a cerebrovascular accident—a loss of neurological function (loss or slurring of speech, altered state of consciousness) caused by an ischemic event and confirmed using either computed tomography or magnetic resonance imaging—or transient neurological deficit, recorded in patients who exhibit a brief episode of neurological dysfunction resulting from focal cerebral ischemia not associated with permanent cerebral infarction.

5. The renal malperfusion cohort includes patients with acute renal failure as defined according to Risk, Injury, Failure, Loss of kidney function, and End-stage kidney disease (RIFLE) criteria.

6. Last, mesenteric malperfusion is reported directly as mesenteric ischemia by centers in patients with evidence of decreased perfusion through the superior mesenteric and inferior mesenteric arteries with decreased viability or necrosis of the gut, with or without lactic acidosis, pain, or abdominal distension.
