# Supplementary material for: Machine learning-based prediction of 1-year mortality using nutritional and inflammatory factors for type A acute aortic dissection with malperfusion
Source: Front Cardiovasc Med. 2025 Sep 29;12:1539267. doi: 10.3389/fcvm.2025.1539267 (PMC12515875; doi:10.3389/fcvm.2025.1539267)
Supplement: Supplementary file 2 [file Datasheet2.pdf]

Supplementary material 2

Inflammatory Indexes

1. **Neutrophil to Lymphocyte Ratio (NLR):**
   NLR is calculated as the ratio of neutrophil count to lymphocyte count, expressed as:

$$NLR = \frac{Neutrophil\ count\ (10^9/L)}{Lymphocyte\ count\ (10^9/L)}$$

This ratio serves as a marker of systemic inflammation, with higher values typically associated with increased inflammatory activity.

2. **Monocyte to Lymphocyte Ratio (MLR):**
   MLR is calculated using the monocyte count divided by the lymphocyte count:

$$MLR = \frac{Monocyte\ count\ (10^9/L)}{Lymphocyte\ count\ (10^9/L)}$$

This index is utilized to evaluate inflammatory responses, particularly in the context of immune system modulation.

3. **Platelet to Lymphocyte Ratio (PLR):**
   PLR is determined by dividing the platelet count by the lymphocyte count:

$$PLR = \frac{Platelet\ count\ (10^9/L)}{Lymphocyte\ count\ (10^9/L)}$$

PLR is a marker of both inflammation and thrombosis, with elevated levels indicating a heightened inflammatory state.

4. **Systemic Immune Inflammation Index (SII):**
   SII is calculated using the following formula:

$$SII = \frac{Platelet\ count\ (10^9/L) \times Neutrophil\ count\ (10^9/L)}{Lymphocyte\ count\ (10^9/L)}$$

SII integrates platelet, neutrophil, and lymphocyte counts to provide a comprehensive assessment of systemic immune-inflammation.

5. **Systemic Inflammation Response Index (SIRI):**
   SIRI is calculated as:

$$SIRI = \frac{Neutrophil\ count\ (10^9/L) \times Monocyte\ count\ (10^9/L)}{Lymphocyte\ count\ (10^9/L)}$$

SIRI combines neutrophil, monocyte, and lymphocyte counts to reflect the systemic inflammatory response.

Nutritional Indexes

1. **Prognostic Nutritional Index (PNI):**
   PNI is calculated to assess the nutritional and immune status of patients, based on serum albumin and lymphocyte count:

$$PNI = 10 \times Serum\ Albumin\ (g/dL) + 0.005 \times Lymphocyte\ (10^9/L)$$

Lower PNI scores are indicative of poor nutritional status and immune competence.

2. **CONUT (Controlling Nutritional Status) Score:**
   The CONUT score is used to evaluate nutritional status based on serum albumin, total cholesterol (TC), and total lymphocyte count (TLC), and is calculated as the sum of individual scores for each component:
   - **Serum Albumin Score:**
     - 0 points: Serum albumin ≥ 3.5 g/dL
     - 2 points: Serum albumin 3.0–3.49 g/dL
     - 4 points: Serum albumin 2.50–2.99 g/dL

        - 6 points: Serum albumin < 2.50 g/dL
    - **Total Cholesterol Score:**
        - 0 points: TC ≥ 180 mg/dL
        - 1 point: TC 140–179 mg/dL
        - 2 points: TC 100–139 mg/dL
        - 3 points: TC < 100 mg/dL
    - **Total Lymphocyte Count Score:**
        - 0 points: TLC ≥ 1.6 × 10^9/L
        - 1 point: TLC 1.20–1.59 × 10^9/L
        - 2 points: TLC 0.80–1.19 × 10^9/L
        - 3 points: TLC < 0.8 × 10^9/L

The final CONUT score is the sum of the points assigned to each component, where higher scores suggest a poorer nutritional status.

3. **Instant Nutritional Assessment (INA) Grading:**
   INA grading is based on the combination of serum albumin levels and total lymphocyte count, and is classified into four grades:

    - **Grade 1:** Serum albumin ≥ 3.5 g/dL, TLC ≥ 1.5 × 10^9/L
    - **Grade 2:** Serum albumin ≥ 3.5 g/dL, TLC < 1.5 × 10^9/L
    - **Grade 3:** Serum albumin < 3.5 g/dL, TLC ≥ 1.5 × 10^9/L
    - **Grade 4:** Serum albumin < 3.5 g/dL, TLC < 1.5 × 10^9/L

This grading system reflects the combined inflammatory and nutritional status, with higher grades indicating more severe impairment in both nutritional and immune function.
