# Supplementary material for: Machine learning-based prediction of 1-year mortality using nutritional and inflammatory factors for type A acute aortic dissection with malperfusion
Source: Front Cardiovasc Med. 2025 Sep 29;12:1539267. doi: 10.3389/fcvm.2025.1539267 (PMC12515875; doi:10.3389/fcvm.2025.1539267)
Supplement: Supplementary file 3 [file Table1.docx]

**Supplementary Table 1 Baseline characteristics of patients**

|  | **Overall**  **(n = 433)** | **Survivors**  **(n = 352)** | **Non-survivors**  **(n = 81)** | **P-Value** |
| --- | --- | --- | --- | --- |
| ***Medical History*** |  |  |  |  |
| Atherosclerosis Of The Aorta, n (%) |  |  |  | 0.04 |
| No | 411 (94.92) | 338 (96.02) | 73 (90.12) |  |
| Yes | 22 (5.08) | 14 (3.98) | 8 (9.88) |  |
| ***Lab*** |  |  |  |  |
| D-dimer (ug/mL), Median (IQR) | 14.74 (8.58-21.07) | 14.64 (7.87-20.99) | 15.66 (10.00-21.70) | 0.14 |
| Troponin I (ng/mL), Median (IQR) | 0.04 (0.02-0.05) | 0.04 (0.02-0.05) | 0.05 (0.03-0.06) | 0.37 |
| ***Complication*** |  |  |  |  |
| Brachiocephalic Artery Occlusion, n (%) |  |  |  | 0.36 |
| No | 383 (88.45) | 309 (87.78) | 74 (91.36) |  |
| Yes | 50 (11.55) | 43 (12.22) | 7 (8.64) |  |
| Cardiac Tamponade, n (%) |  |  |  | 0.19 |
| No | 391 (90.30) | 321 (91.19) | 70 (86.42) |  |
| Yes | 42 (9.70) | 31 (8.81) | 11 (13.58) |  |
| Neurological Deficit, n (%) |  |  |  | 0.77 |
| No | 399 (92.15) | 325 (92.33) | 74 (91.36) |  |
| Yes | 34 (7.85) | 27 (7.67) | 7 (8.64) |  |
| Severe Aortic Regurgitation, n (%) |  |  |  | <0.01 |
| No | 388 (89.61) | 340 (96.59) | 48 (59.26) |  |
| Yes | 45 (10.39) | 12 (3.41) | 33 (40.74) |  |
| Shock, n (%) |  |  |  | 0.02 |
| No | 388 (89.61) | 321 (91.19) | 67 (82.72) |  |
| Yes | 45 (10.39) | 31 (8.81) | 14 (17.28) |  |
| ***Other*** |  |  |  |  |
| Time From Symptom Onset To Surgery (hours), Median (IQR) | 4 (2 - 6) | 4 (2 - 6) | 7 (4 -10) | <0.01 |
|  |  |  |  |  |
