# Supplementary figures and images for: Machine learning-based prediction of 1-year mortality using nutritional and inflammatory factors for type A acute aortic dissection with malperfusion
Source: Front Cardiovasc Med. 2025 Sep 29;12:1539267. doi: 10.3389/fcvm.2025.1539267 (PMC12515875; doi:10.3389/fcvm.2025.1539267)

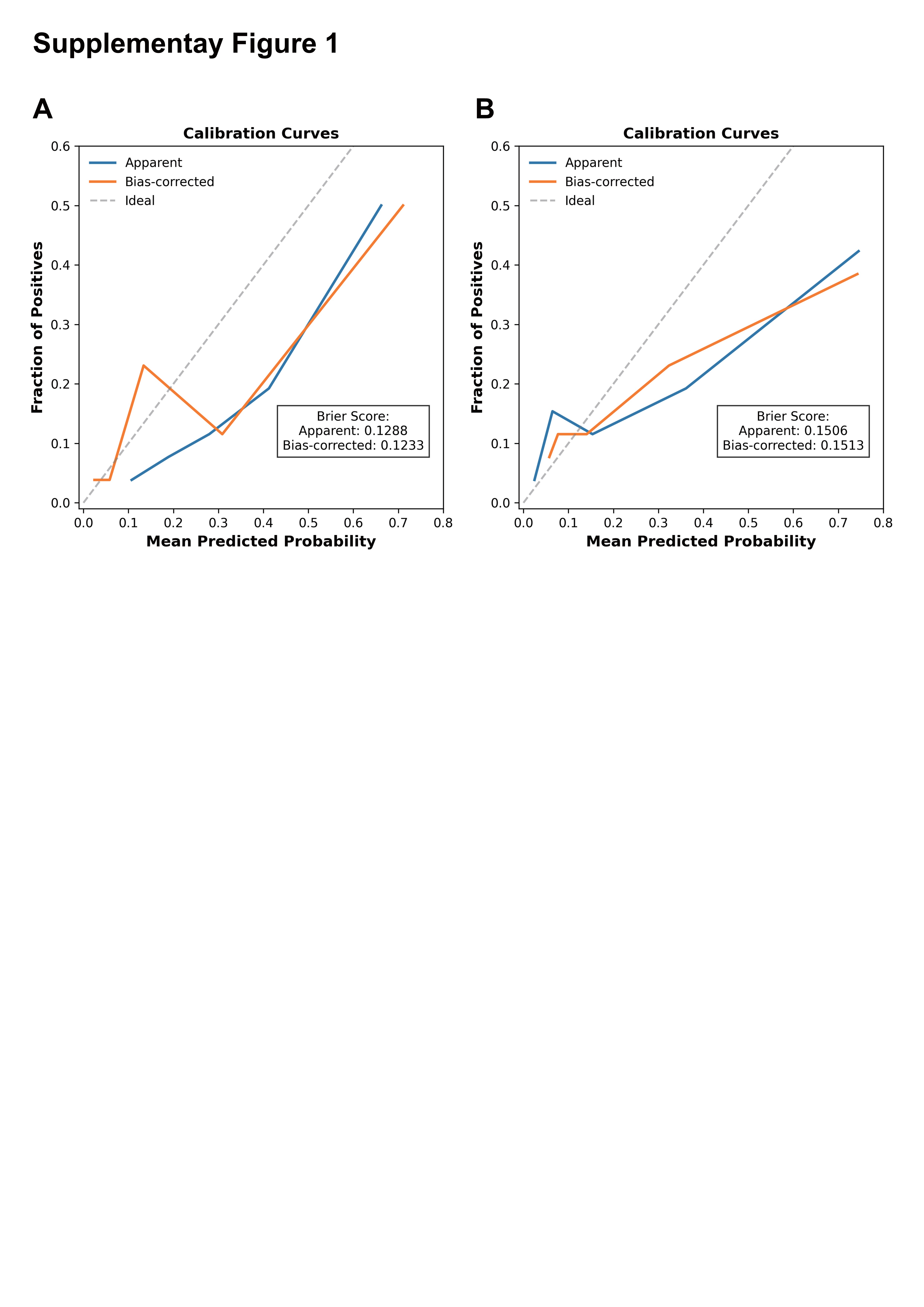

Supplement: Supplementary Figure S1. — Calibration curves for (A) the Random Forest model using inflammatory and nutritional laboratory values and (B) the XGBoost model using inflammatory and nutritional indices. The dashed line (Ideal) represents perfect calibration, where predicted probabilities exactly match observed outcomes. The blue solid line (Apparent) shows the observed performance of the model, while the yellow solid line (Bias-corrected) reflects bias-adjusted performance. [file Image1.jpeg]
